# Impact of smoking on immune feature and prognosis in unresectable stage III anaplastic lymphoma kinase positive non-small-cell lung cancer

**DOI:** 10.3389/fonc.2025.1594479

**Published:** 2026-01-23

**Authors:** Ying Jiang, Zhihui Zhang, Jianzhong Cao, Jianchun Duan, Tao Zhang, Yu Wang, Weihua Li, Fengwei Tan, Jianming Ying, Nan Bi

**Affiliations:** 1 Department of Radiation Oncology, National Cancer Center/National Clinical Research Center for Cancer/Cancer Hospital, Chinese Academy of Medical Sciences and Peking Union Medical College, Beijing, China; 2 Department of Radiation Oncology, The Shanxi Province Cancer Hospital/Shanxi Hospital Affiliated to Cancer Hospital, Chinese Academy of Medical Sciences/Cancer Hospital Affiliated to Shanxi Medical University, Taiyuan, Shanxi, China; 3 CAMS Key Laboratory of Translational Research on Lung Cancer, State Key Laboratory of Molecular Oncology, Department of Medical Oncology, National Cancer Center/National Clinical Research Center for Cancer/Cancer Hospital, Chinese Academy of Medical Sciences, Peking Union Medical College, Beijing, China; 4 Department of Respiratory Medicine, Shanxi Province Cancer Hospital/Shanxi Hospital Affiliated to Cancer Hospital, Chinese Academy of Medical Sciences/Cancer Hospital Affiliated to Shanxi Medical University, Taiyuan, Shanxi, China; 5 Department of Pathology, State Key Laboratory of Molecular Oncology, National Cancer Center/National Clinical Research Center for Cancer/Cancer Hospital, Chinese Academy of Medical Sciences and Peking Union Medical College, Beijing, China; 6 Department of Thoracic Surgery, National Cancer Center/National Clinical Research Center for Cancer/Cancer Hospital, Chinese Academy of Medical Sciences and Peking Union Medical College, Beijing, China

**Keywords:** unresectable, non-small-cell lung cancer, anaplastic lymphoma kinase, radiotherapy, systemic therapy

## Abstract

**Introduction:**

Smoking is the primary risk factor for lung cancer, and 37% - 42% of patients with non-small-cell lung cancer (NSCLC) harboring anaplastic lymphoma kinase (*ALK*) mutation being smokers. Nevertheless, the specific impact of smoking on prognosis in patients with unresectable stage III *ALK*-positive NSCLC remains to be elucidated.

**Method:**

This two-centric, retrospective cohort study included 48 patients with unresectable stage III *ALK*-positive NSCLC. Gene ontology (GO) enrichment analysis was conducted on data from 25 patients who underwent NGS. We further performed Gene Set Enrichment Analysis (GSEA) and Kyoto Encyclopedia of Genes and Genomes (KEGG) pathway analysis to validate these findings, using the GSE31852 dataset (n = 34) patients from the Gene Expression Omnibus (GEO) database.

**Results:**

In these 48 patients, the median age was 55.2 (range, 33-80) years; approximately half of the patients were men (50.0%) and smokers (45.8%); 62.5% patients had IIIB stage disease; 33.3% patients initially received chemoradiation therapy (CRT). After a median follow-up of 49.02 (interquartile range [IQR], 35.84 - 62.03) months, CRT significantly improved the locoregional-free survival (LRFS, *P* = 0.012). Univariate and multivariate Cox regression analysis suggested that smoking was independent prognostic factors for poorer OS (univariate HR = 3.01, *P* = 0.049; multivariate HR = 3.92, *P* = 0.023). Compared with never-smokers, smokers exhibited a significantly inferior 5-year OS (51.9% vs. 78.9%, Log-rank *P* = 0.038). GO analysis revealed distinct biological processes and cell components between never-smokers and smokers. Validation in the GSE31852 dataset subsequently confirmed these findings and further highlighted the significant differences in immune cell regulation, including immune cell infiltration, differentiation, and interactions between never-smokers and smokers.

**Conclusions:**

In patients with unresectable stage III *ALK*-positive NSCLC, CRT improved the disease control. Smokers exhibited a significantly poorer OS and DMFS, and may require more risk-adapted treatment strategies, such as the combination of CRT with upfront *ALK* TKIs. These findings suggest that smoking may adversely affect survival by modulating the tumor immune microenvironment.

## Introduction

Lung cancer is the leading cause of death worldwide, with non-small-cell lung cancer (NSCLC) accounting for 85% of all lung cancer (1). Anaplastic lymphoma kinase (*ALK*) rearrangements are found in approximately 5% of NSCLC (2). The proportion of smokers among patients with *ALK*-positive NSCLC is slightly higher than that among those with *EGFR*-mutated NSCLC, although approximately 37–42% of *ALK*-positive patients are current or former smokers (3–11). Previous molecular epidemiology studies have demonstrated distinct mutation patterns and frequencies between smokers and never-smokers with NSCLC, including differences in *TP53*, *KRAS*, and *EGFR* mutations (12, 13). It is therefore important to identify the impact of smoking on unresectable stage III *ALK*-positive NSCLC.

The introduction of *ALK* tyrosine kinase inhibitors (TKIs) has substantially improved survival in advanced-stage disease. Smoking status does not appear to significantly efficacy of first-line treatment (14). Although *ALK* TKIs have been evaluated in resectable disease in several recent clinical trials (15, 16), evidence in unresectable stage III *ALK*-positive NSCLC remains limited. Currently, chemoradiotherapy (CRT) and immunotherapy remain the standard treatment for patients with unresectable stage III NSCLC who have good performance status (17). However, due to exclusion of *ALK*-positive NSCLC in the majority of clinical trials and poor outcomes of immunotherapy in *ALK*-positive NSCLC, the optimal treatment for stage III *ALK*-positive unresectable NSCLC remains unclear. The clinical characteristics and prognosis of these patients have been rarely reported, leaving limited evidence to guide clinical practice.

In this study, we aimed to evaluate the clinical outcomes and the impact of smoking on unresectable stage III *ALK*-positive NSCLC.

## Methods

### Patients

This two-centric, retrospective cohort study included patients who met the following criteria: pathologically unresectable stage III NSCLC; *ALK*-positive status was identified by one of Ventana D5F3 immunohistochemistry (IHC), fluorescent *in situ* hybridization (FISH), reverse transcription-polymerase chain reaction (RT-PCR) or next-generation sequencing (NGS); and adequate follow-up data. The follow-up period ranged from the date of diagnosis to either the last follow-up or death. Participants were followed every 6 months through clinic visits or telephone interviews.

Patients were categorized into two treatment groups: the definitive radiation therapy (RT) group and the systemic therapy group. Definitive RT was defined as volumetric modulated arc therapy (VMAT) or intensity-modulated radiation therapy (IMRT) with a prescribed dose of 60–70 Gy, delivered sequentially or concurrently with at least two cycles of platinum-based doublet chemotherapy. The systemic therapy group comprised patients who were unsuitable for definitive RT or those who refused it, opting for drug therapy as their initial treatment.

### Detection of gene mutation and identification of *ALK* variant

NGS was used to detect the *ALK* rearrangements. *EML4*-*ALK* v3 was defined as the fusion of exon 6a/b in *EML4* to exon 20 in *ALK*, while non-*EML4-ALK* v3 variants included other *EML4–ALK* fusions (e.g., *EML4–ALK* v1, v2) and additional *ALK* fusion partners such as *TMEM178A*-*ALK* and *KCNK2*-*ALK*. Co-mutation was defined as *ALK*-positive status combined with other mutation, such as *EGFR* and BRCA2. Gene ontology (GO) enrichment analysis was performed on data from 25 patients who underwent NGS.

### Enrichment analysis

GO enrichment analysis was performed to investigate the cellular functions of smoking-related genes. Smoking-related genes were defined as differentially expressed genes between smokers and never-smokers in the GSE31852 cohort, with an absolute log2 fold change (|log2FC|) > 2 and P < 0.05, yielding 89 genes. The Kyoto Encyclopedia of Genes and Genomes (KEGG) pathway enrichment analysis was performed. Analysis was conducted using the online Database for Annotation, Visualization, and Integrated Discovery (DAVID 6.8, https://david.ncifcrf.gov/) to explore the potential biological roles of smoking-related genes. Gene set variation analysis (GSVA) was performed using R 3.5.1 to estimate variation in gene set enrichment according to the expression data, using the GSE31852 dataset (n = 34) from the Gene Expression Omnibus (GEO) database. The GSVA package was freely available at http://www.bioconductor.org.

### Statistical analysis

Progression-free survival (PFS), locoregional-free survival (LRFS), distant metastasis-free survival (DMFS), and overall survival (OS) were defined as the time from diagnosis to disease progression or death, locoregional recurrence or death, distant recurrence or death, and death, respectively. Kaplan–Meier curves were used to estimate survival outcomes. Baseline characteristics between the groups were compared using the Chi-square test or Fisher’s exact test for categorical variables, as appropriate. Landmark analysis was employed to reduce the impact of long-term events on outcomes. Univariate and multivariate regression analyses were carried out using a Cox proportional hazards model, and covariates with P<0.2 in the univariate analysis were included in the multivariate analysis. All statistical analyses were performed using R software (version 4.3.2), with two-sided P < 0.05 was considered statistically significant.

## Results

### Patients characteristics

A total of 48 patients with stage III unresectable *ALK*-positive NSCLC met the eligibility criteria and were included in the analysis (**Supplementary Figure S1**). The baseline characteristics of patients are summarized in **Table 1**. The median age was 55.2 (33–80) years; almost half of the patients were men (50.0%) and current or former smokers (45.8%); 62.5% had stage IIIB disease; most patients (93.8%) had adenocarcinoma; all patients had an Eastern Cooperative Oncology Group performance status (ECOG PS) of 0-1. 16 (33.3%) patients received definitive CRT as the initial treatment; 14 (29.2%) patients had *EML4*-*ALK* v3 status. Regarding treatment-related toxicities, pneumonitis was observed in 6/16 patients in the RT group, including one case of grade 3 case.

**Table 1:** Baseline characteristics.

Characteristics	*N=48*
Age (range)	55.2 (33-80)
Sex:	
Woman	24 (50.0%)
Man	24 (50.0%)
ECOG PS:	
0	20 (41.7%)
1	28 (58.3%)
Smoking:	
No	26 (54.2%)
Yes	22 (45.8%)
Stage:	
IIIA	12 (25.0%)
IIIB	30 (62.5%)
IIIC	6 (12.5%)
Histological type:	
Adenocarcinoma	45 (93.8%)
Non-adenocarcinoma	3 (6.25%)
*EML4*-*ALK* variants.:	
V3	14 (29.2%)
Others	14 (29.2%)
Unknown	20 (41.7%)
Treatment:	
Chemoradiation therapy	16 (33.3%)
Sequential chemoradiation therapy	6 (12.5%)
Concurrent chemoradiation therapy	10 (20.8%)
Systemic therapy	32 (66.7%)

ECOG PS, Eastern Cooperative Oncology Group performance status; *ALK*, anaplastic lymphoma kinase; TKIs, tyrosine kinase inhibitors.

### Survival analysis and first failure pattern

**Figure 1:**
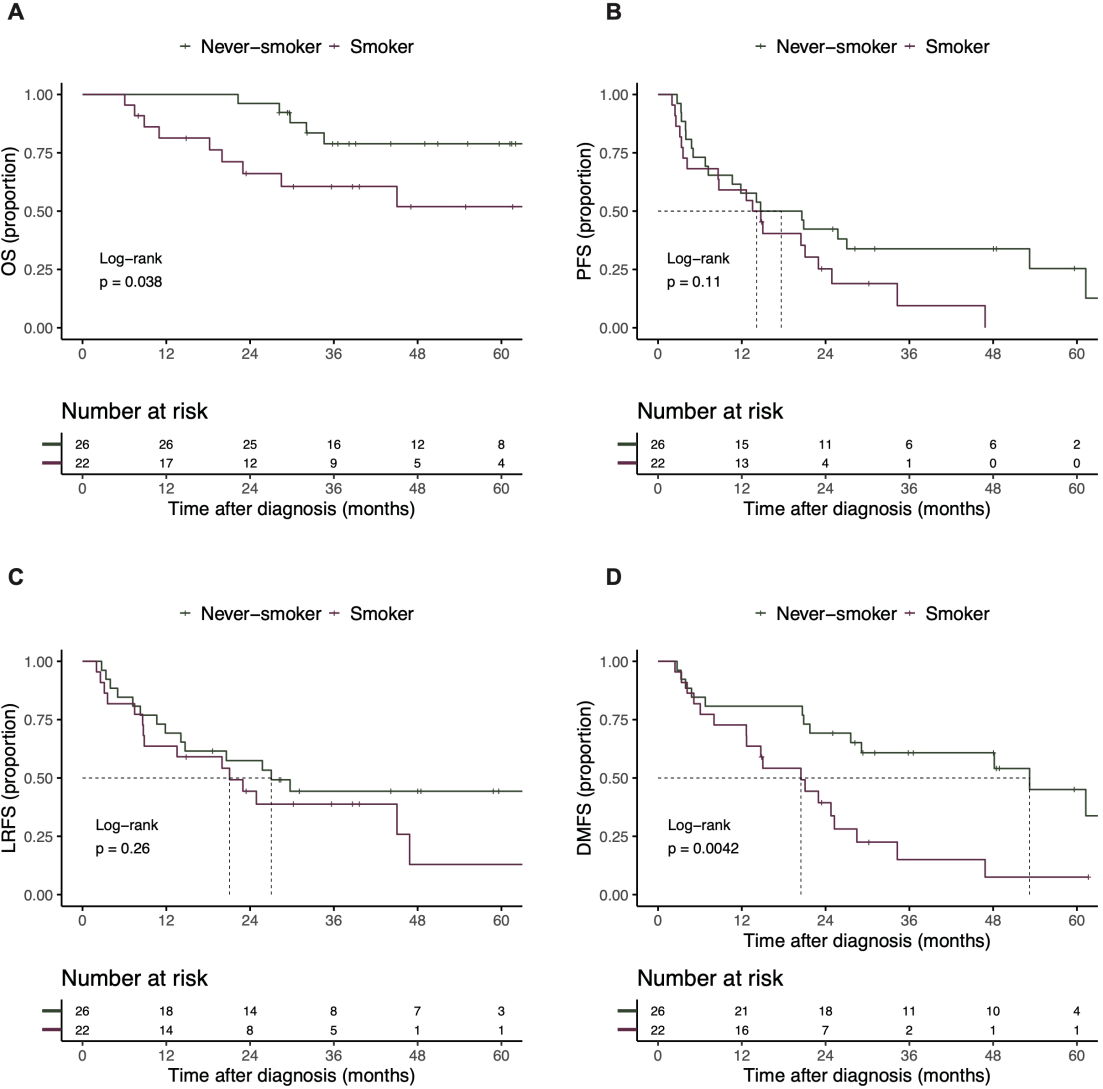
Kaplan-Meier curves for the OS **(A)**, PFS **(B)**, LRFS **(C)** and DMFS **(D)** between CRT group and systemic therapy group. OS, overall survival; PFS, progression-free survival; LRFS, locoregional free survival; DMFS, distant metastasis-free survival; CRT, chemoradiation therapy.

**Figure 2:**
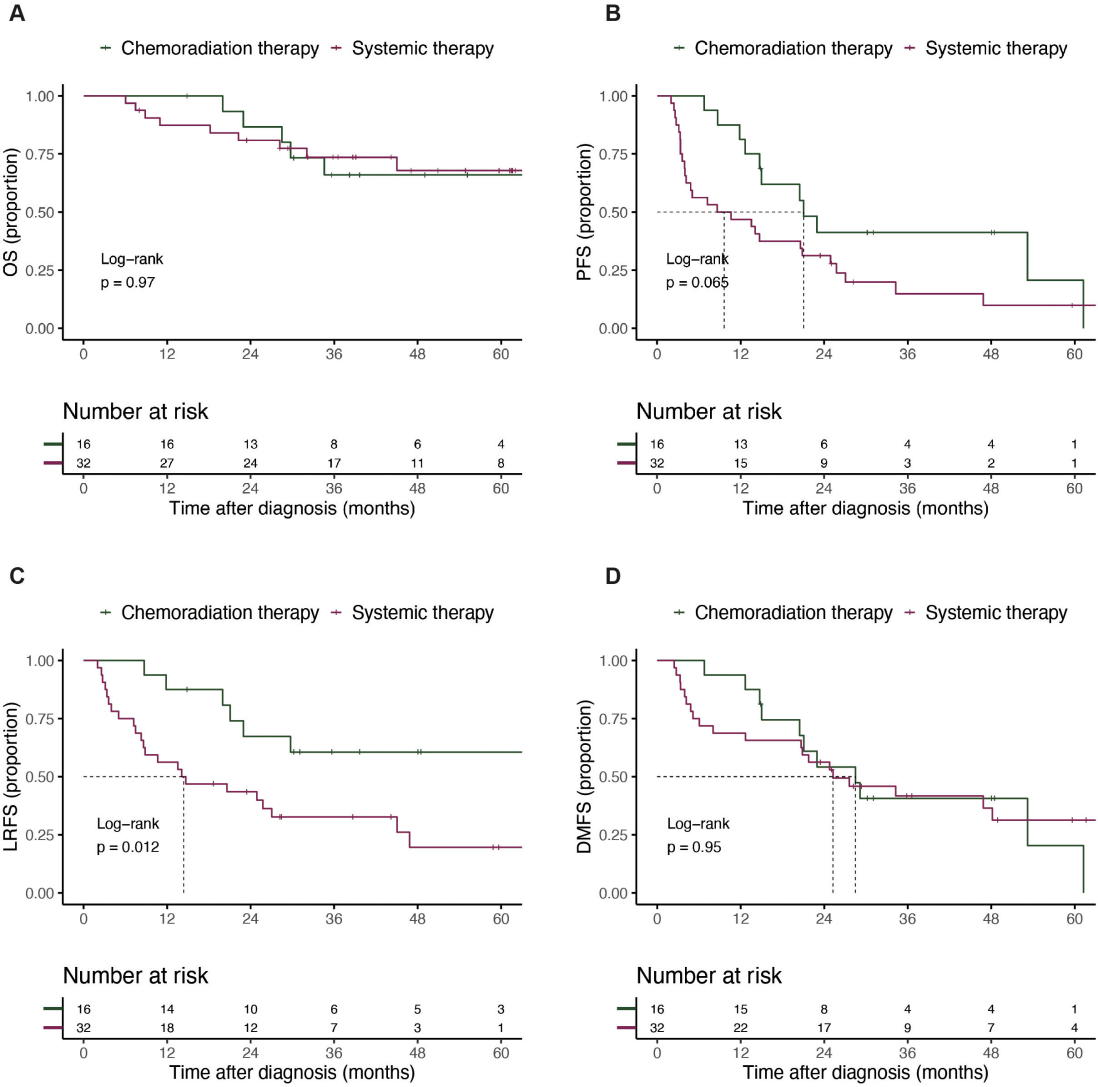
Kaplan-Meier curves for the OS **(A)**, PFS **(B)**, LRFS **(C)** and DMFS **(D)** between never-smokers group and smokers group. OS, overall survival; PFS, progression-free survival; LRFS, locoregional free survival; DMFS, distant metastasis-free survival.

The median follow-up time for the entire cohort was 49.02 (interquartile range [IQR], 35.84 -62.03) months. The 1-, 3-, 5-year OS, and median OS for all patients were 91.6% (95% confidence interval [CI]: 84.0% - 99.8%), 70.7% (95% CI: 58.4% - 85.6%), 67.0% (53.8% - 83.4%) and not reached, respectively; the 1-, 3-, 5-year PFS, and median PFS was 58.3% (95% CI: 45.9% - 74.1%), 24.1% (95% CI: 14.0% - 41.4%), 15.1% (95% CI: 6.7% - 35.8%) and 14.7 months (95% CI: 10.6-24.9), respectively (**Supplementary Figure S2**). Compared with systemic therapy, the Kaplan-Meier curves showed that CRT improved the mPFS (21.1 vs. 9.6 months, P = 0.065; **Figure 1B**) and mLRFS (NA vs. 14.4 months, P = 0.012; **Figure 1C**). In contrast, no significant difference was observed in OS or DMFS between the CRT and systemic therapy. Subsequently, we examined the effect of smoking status on OS, PFS, LRFS, and DMFS (**Figure 2**). The 5-year OS rate was significantly higher in the never-smokers group at 78.9% (95% CI: 63.9% - 97.3%) compared to 51.9% (95% CI: 32.6% - 82.8%) in the smokers group (P = 0.038; **Figure 2A**). Similar trends were observed for DMFS, with a 5-year DMFS of 45.0% (95% CI: 26.6% - 76.4%) versus 7.5% (95% CI: 1.2% - 45.6%) in the never-smokers and smokers groups, respectively (P = 0.004; **Figure 2D**).

As summarized in **Supplementary Table S2**, 77.1% of patients had relapsed. 15 (31.3%) patients had distant recurrence only and 13 (27.1%) patients had locoregional and distant recurrence simultaneously. Brain (14/28) was the most common metastasis site. Compared with systemic therapy, CRT significantly decreased the locoregional recurrence rate (18.8% vs. 59.4%, P = 0.018, **Supplementary Table S2**).

### Univariate and multivariate Cox regression analysis for PFS and OS

The univariate Cox regression analysis revealed that smoking status (hazard ratio [HR] = 3.01, 95% CI: 1.01 - 9.02, P = 0.049) and stage (IIIB vs. IIIA, HR = 0.18, 95% CI: 0.06 - 0.53, P = 0.002) were significantly associated with OS (**Table 2**). In the univariate Cox regression analysis, the variable stage IIIC produced an infinite HR due to the absence of observed deaths in this category, which this variable was excluded from the multivariate Cox regression analysis to ensure model stability and reliability. In the multivariate Cox regression analysis, after adjusting for confounders, smoking status (HR = 3.92, 95% CI: 1.21 - 12.73, P = 0.023) remained a significant predictor of OS (**Table 2**), indicating smoking status was the independent prognostic factor for OS in stage III *ALK*-positive NSCLC. As for PFS (**Table 3**), univariate COX analysis suggested that patients who received systemic therapy tended to have a poorer PFS (HR = 1.92, 95%CI 0.95 - 3.89 *P* = 0.069). However, after adjusting for potential confounders (Covariates with P<0.2 in the univariate analysis) in the multivariate analysis, treatment was found to be significantly associated with PFS (aHR: 3.04, 95% CI: 1.32 - 7.01, p = 0.009), which indicates CRT exerts a significant effect on PFS when accounting for other covariates.

**Table 2:** Univariate and multivariate analysis to evaluate the association between prognostic factors and overall survival.

Characteristics	Univariate analysis			Multivariate analysis*		
	HR	CI	*P*	aHR	CI	*P*
Age						
	1			1		
	0.43	0.12 – 1.54	0.193	0.47	0.11 – 2.02	0.313
ECOG PS						
0	1					
1	1.06	0.35 – 3.16	0.919			
Smoker						
No	1			1		
Yes	3.01	1.01 – 9.02	0.049	3.92	1.21 - 12.73	0.023
Stage						
IIIA	1					
IIIB	0.18	0.06 – 0.53	0.002			
IIIC	0	0 – Inf				
Treatment						
CRT	1			1		
Systemic therapy	0.98	0.33 – 2.92	0.969	1.16	0.36 – 3.73	0.801
EML4-ALK						
Others	1					
V3	2.66	0.52 – 13.73	0.244	1.94	0.35 – 10.79	0.447
Unknown	3.73	0.77 – 18.10	0.102	3.05	0.53 – 17.40	0.211

ECOG PS, Eastern Cooperative Oncology Group performance status; HR, hazard ratio; CI, confidence interval.

*Covariates with P<0.2 in the univariate analysis and treatment were included in the multivariate analysis. In the univariate Cox regression analysis, the variable stage yielded an infinite HR due to the absence of observed deaths in this category. Consequently, this variable was excluded from the multivariate Cox regression analysis to ensure model stability and reliability.

**Table 3:** Univariate and multivariate analysis to evaluate the association between prognostic factors and progression-free survival.

Characteristics	Univariate analysis			Multivariate analysis*		
	HR	CI	*P*	aHR	CI	*P*
Age						
	1					
	1.24	0.65 – 2.36	0.515			
ECOG PS						
0	1					
1	0.78	0.41 - 1.49	0.451			
Smoker						
No	1					
Yes	1.72	0.88 – 3.33	0.111	1.64	0.83 - 3.24	0.154
Stage						
IIIA	1					
IIIB	0.55	0.27 – 1.15	0.113	0.38	0.16 – 0.91	0.029
IIIC	0.78	0.27 – 2.28	0.653	0.38	0.11 – 1.31	0.125
Treatment						
CRT	1					
Systemic therapy	1.92	0.95 – 3.89	0.069	3.04	1.32 – 7.01	0.009
EML4-ALK						
Others	1					
V3	1.44	0.62 – 3.36	0.393			
Unknown	1.49	0.67 – 3.33	0.332			

ECOG PS, Eastern Cooperative Oncology Group performance status; HR, hazard ratio; CI, confidence interval.

*Covariates with P<0.2 in the univariate analysis were included in the multivariate analysis.

### GO and pathway enrichment analysis in smokers vs. non-smokers

To further investigate the differences between smokers and never-smokers, we performed GO analyses. As illustrated in **Supplementary Figure S3**, the results revealed distinct revealed distinct enrichment patterns. For biological processes (BP), never-smokers exhibited enrichment in peptidyl-tyrosine modification, positive regulation of kinase activity, and peptidyl-tyrosine phosphorylation, whereas smokers showed enrichment in gland development, epithelial cell proliferation, and regulation of the mitotic cell cycle. In terms of cellular components (CC), never-smokers were enriched in transcription repressor complex, transcription regulator complex, and PML body, whereas smokers were enriched in chromosome, telomeric region, DNA repair complex, and PML body. For molecular functions (MF), both smokers and never-smokers demonstrated enrichment in transmembrane protein kinase activity, protein serine/threonine/tyrosine kinase activity, and transmembrane receptor protein tyrosine kinase activity. Additionally, KEGG pathway analyses indicated that smokers were particularly enriched in pathways related to cellular senescence.

**Figure 3:**
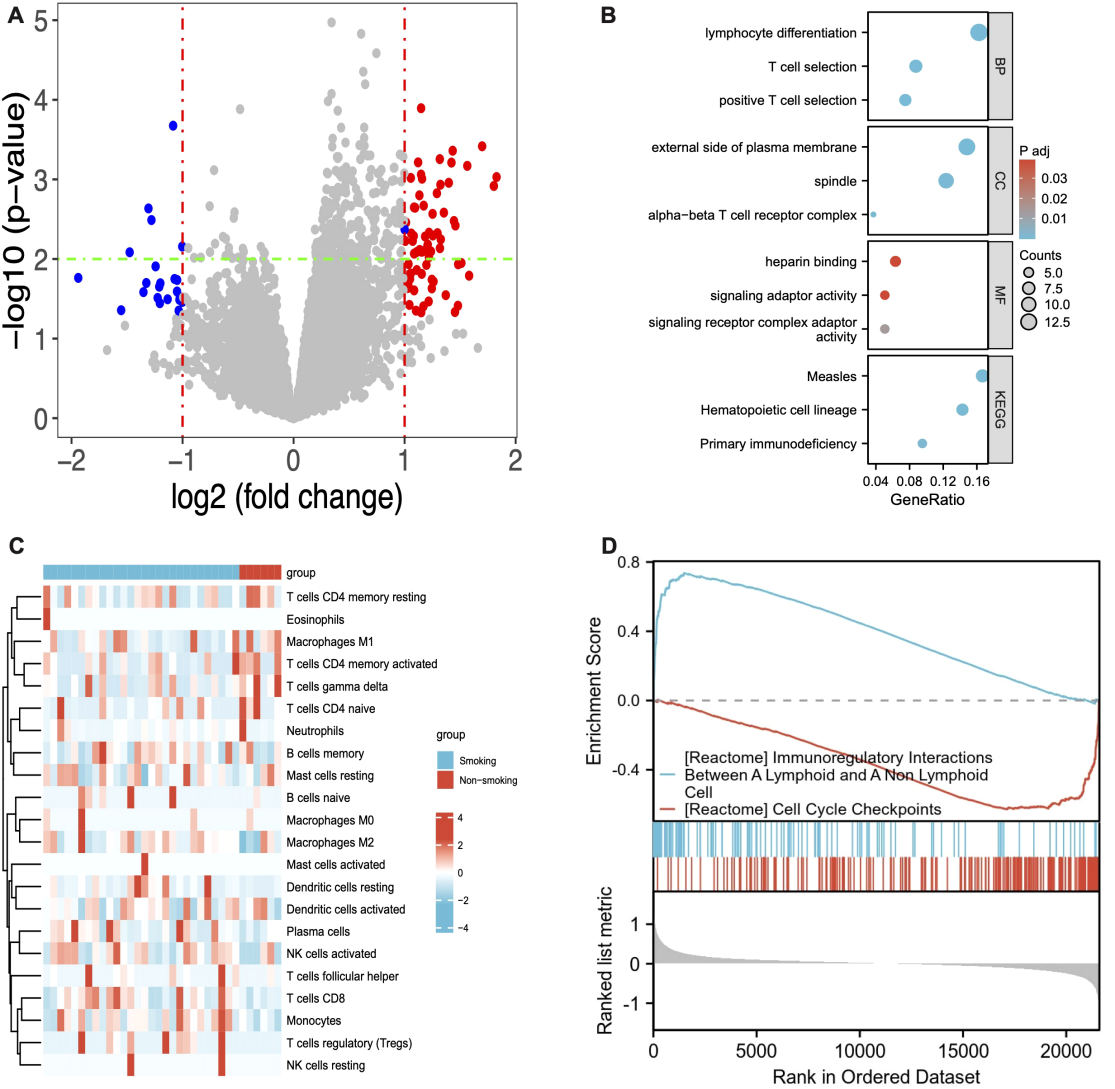
Volcano plot **(A)** and Heatmap **(C)** showing the DEGs between smokers and never-smokers; GO **(B)** and KEGG **(D)** enrichment analysis for the smokers. DEGs, differentially expressed genes; GO, Gene Ontology; KEGG, Kyoto Encyclopedia of Genes and Genomes.

The GSE31852 cohort was used for subsequent analysis to explore the differences between smokers and never-smokers. Initially, 89 differentially expressed genes were identified. **Figure 3B** shows a heatmap presenting the detailed expression of smoking-related genes. GO analysis indicated that these genes were enriched in lymphocyte differentiation, T cell selection, and positive T cell selection in the BP category; external side of plasma membrane, spindle, and alpha-beta T cell receptor complex in the CC category; and heparin binding and signaling receptor complex adaptor activity in the MF category. KEGG enrichment analysis revealed that these smoking-related genes might be associated with measles, hematopoietic cell lineage, and primary immunodeficiency (**Figure 3C**). To gain a deeper understanding of the mechanisms underlying these differentially expressed genes, we utilized GSEA and found that the genes were related to immunoregulatory interactions between a lymphoid and a non-lymphoid cell, as well as cell cycle checkpoints.

## Discussion

This study explored the clinical outcomes and impact of smoking on patients with unresectable stage III *ALK*-positive NSCLC. In terms of treatment strategy, CRT can improve disease control. Our findings demonstrate significant differences in the prognosis and molecular characteristics between smokers and never-smokers, indicating smokers may require more risk-adapted treatment strategies, such as the combination of CRT with upfront *ALK* TKIs.

Our data show that the smoking status is a critical prognostic factor for OS in patients with unresectable stage III *ALK*-positive NSCLC. Both univariate and multivariate Cox regression analysis confirmed that smokers had a significantly higher risk of mortality compared with never-smokers, with a HR for overall survival of 3.92. These findings highlight the detrimental impact of smoking on survival outcomes in this population. Several mechanisms may explain the negative prognostic effect of smoking. Tobacco exposure induces widespread genomic instability and increases tumor mutational burden, potentially reducing sensitivity to targeted therapies and chemoradiotherapy (12, 13, 18, 19). The prognostic value of tumor-infiltrating lymphocytes is influenced by smoking, indicating smoking may alter the immune microenvironment (20). Consistent with our findings, previous studies have reported that smokers with advanced *ALK*-positive NSCLC have poorer survival than never-smokers (21). The biological basis of these observations should be further explored to determine whether this might be due to a specific mutation pattern produced by tobacco exposure.

CRT remains the standard treatment for patients with good performance status and unresectable stage III *ALK*-positive NSCLC. Our study found that CRT improved PFS and LRFS compared to systemic therapy, underscoring its importance in this cohort. Importantly, patients in the CRT group were not deprived of targeted therapy; most received ALK TKIs at relapse, and three patients received upfront *ALK* TKI without subsequent recurrence. However, no significant difference in OS or DMFS was observed between the two groups. Considering the robust efficacy of *ALK* TKIs, combining CRT with upfront *ALK* TKIs may be the optimal treatment option, especially in smokers. Recently, the integration of local therapy with targeted agents has shown significant clinical benefits in NSCLC and is changing treatment paradigms. Although the ALINA trial demonstrated that *ALK* TKIs significantly improved PFS in patients with resected early-stage *ALK*-positive NSCLC, its results highlight the potential value of *ALK* inhibition across different disease settings (8). Similarly, the LAURA study demonstrates that *EGFR* TKIs after CRT significantly prolong the PFS compared to placebo in stage III *EGFR*-mutated NSCLC (3). These results indicate that the strategy of local treatment combined with upfront TKIs is highly promising. A meeting abstract retrospectively reported that upfront *ALK* TKI demonstrated clinically meaningful improvement in PFS and OS over consolidation immunotherapy and observation (22). Currently, results from clinical trials investigating CRT combined with upfront *ALK* TKIs have not yet been reported, which necessitates further exploration.

The GO and KEGG enrichment analysis suggested distinct BP, CC and MF between smokers and never-smokers. Non-smokers exhibited enrichment in processes related to peptidyl-tyrosine modification and kinase activity, which are consistent with pathways that are important for cellular signaling and growth regulation. In contrast, smokers showed enrichment in gland development and epithelial cell proliferation, which may potentially contribute to more aggressive tumor phenotypes and poorer outcomes (23). Furthermore, smokers were particularly enriched in pathways related to cellular senescence, a hallmark of aging and cancer progression. Prior studies have shown that the pro-inflammatory senescence-associated secretory phenotype (SASP) can promote tumor progression (24). In our study, however, these enrichment patterns should be interpreted as associative and exploratory rather than causal. These molecular findings generate hypotheses about potential biological differences between smokers and never-smokers in stage III *ALK*-positive NSCLC, but they do not establish a direct causal link between smoking, the altered molecular pathways, and the observed survival differences. The results were then further explored in the GSE31852 cohort. The GO analysis indicated that these genes were enriched in immune-related processes, such as lymphocyte differentiation and T cell selection, suggesting a potential link between smoking, immune dysregulation, and NSCLC progression. The KEGG enrichment analysis further implicated these genes in pathways related to measles, hematopoietic cell lineage, and primary immunodeficiency, highlighting the complex interaction between smoking, immunity, and cancer. The GSEA revealed associations with immunoregulatory interactions and cell cycle checkpoints, which are crucial for maintaining genomic stability and preventing tumor development in general.

Our study has several limitations. First, its retrospective design and the relatively small sample size inevitably restrict statistical power. The limited sample size also prevented further investigation into the potential interplay between smoking status and sex. Second, the systemic therapy group consisted of patients receiving different *ALK* TKI generations or chemotherapy, and this inherent heterogeneity may affect survival comparisons with the CRT group. Given the limited sample size, further stratified analyses were not feasible, and the enrichment results based on the small NGS subset should therefore be regarded as exploratory. Moreover, the external dataset used for molecular validation differed from our cohort in baseline characteristics, and detailed information on treatment history was not available. Therefore, it was used solely to provide an independent molecular comparison between smokers and never-smokers, rather than to replicate clinical outcomes. Finally, given the distinct genomic and immunologic features associated with smoking, further mechanistic studies in larger, prospectively collected cohorts are required to validate and extend these observations.

In conclusion, our study highlights the significant impact of smoking on the prognosis of patients with unresectable stage III *ALK*-positive NSCLC. Smoking status emerges as an independent negative prognostic factor for OS, which should be considered when designing treatment strategies. CRT exhibits advantages in controlling locoregional disease, while systemic therapy may be necessary to address distant metastases. Future prospective studies with larger cohorts are needed to validate our results and further explore the molecular mechanisms underlying the impact of smoking on *ALK*-positive NSCLC.

## Data Availability

The external validation dataset GSE31852 used in this study is publicly available in the Gene Expression Omnibus (GEO) database (https://www.ncbi.nlm.nih.gov/geo/). The clinical datasets generated and analyzed during the current study contain patient-level information and are therefore not publicly accessible due to institutional ethical restrictions. De-identified data may be made available from the corresponding author upon reasonable request and with approval from the institutional ethics committee.
