# Supplementary material for: Impact of smoking on immune feature and prognosis in unresectable stage III anaplastic lymphoma kinase positive non-small-cell lung cancer
Source: Front Oncol. 2026 Jan 23;15:1594479. doi: 10.3389/fonc.2025.1594479 (PMC12875992; doi:10.3389/fonc.2025.1594479)

Supplementary Figure S1. Study Flow Chart.


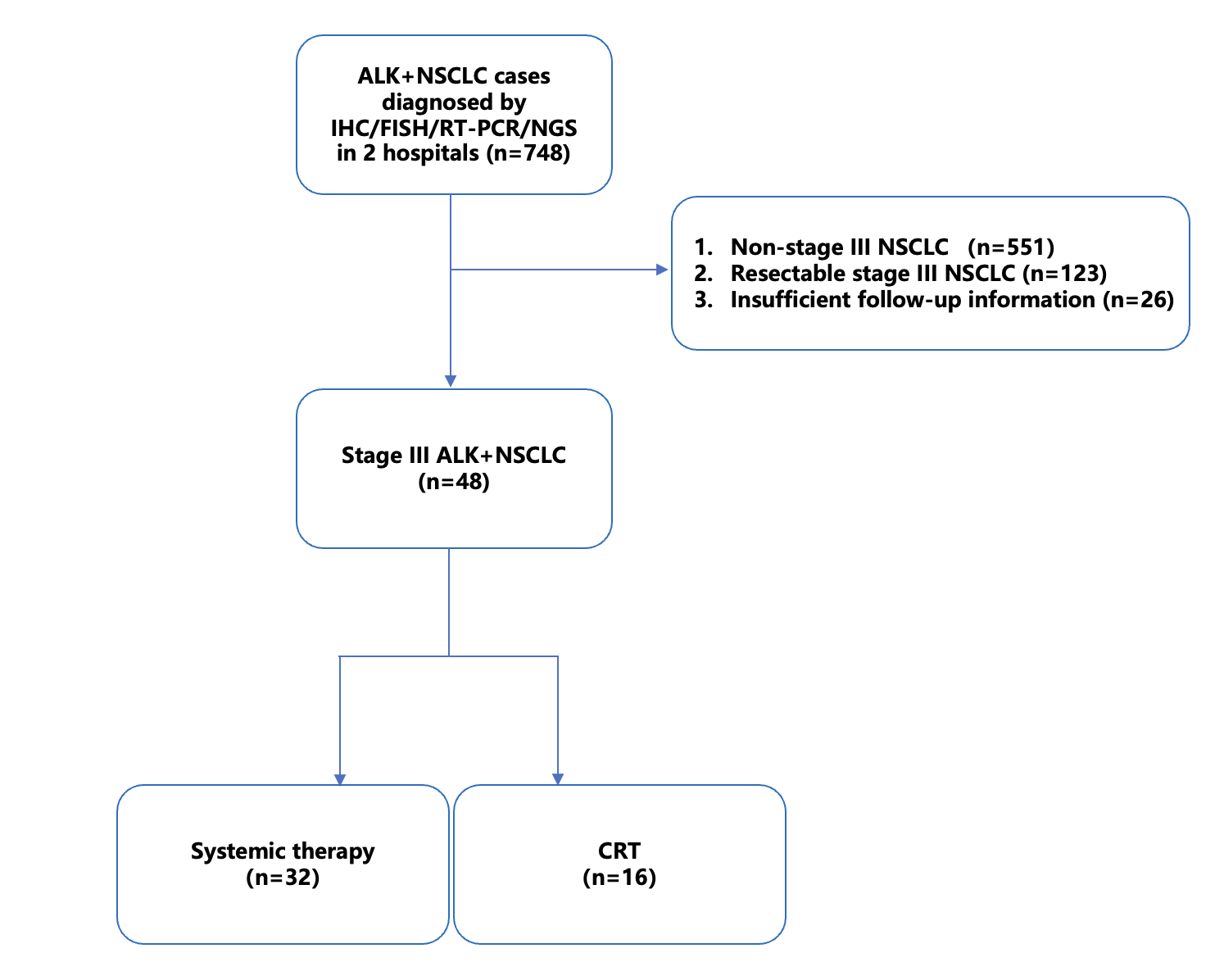


NSCLC, non-small cell lung cancer; IHC, immunohistochemistry; FISH, fluorescent in situ hybridization; RT-PCR, reverse transcription-polymerase chain reaction; NGS, next generation sequencing.

Supplementary Figure S2. Kaplan-Meier curves for the (A) OS and (B) PFS in all patients.

OS, overall survival; PFS, progression-free survival.

Supplementary Figure S3. GO enrichment analysis for the (A) never-smokers and (B) smokers.

GO, Gene Ontology.

Supplementary Figure S4. Venn diagram for first failure pattern.


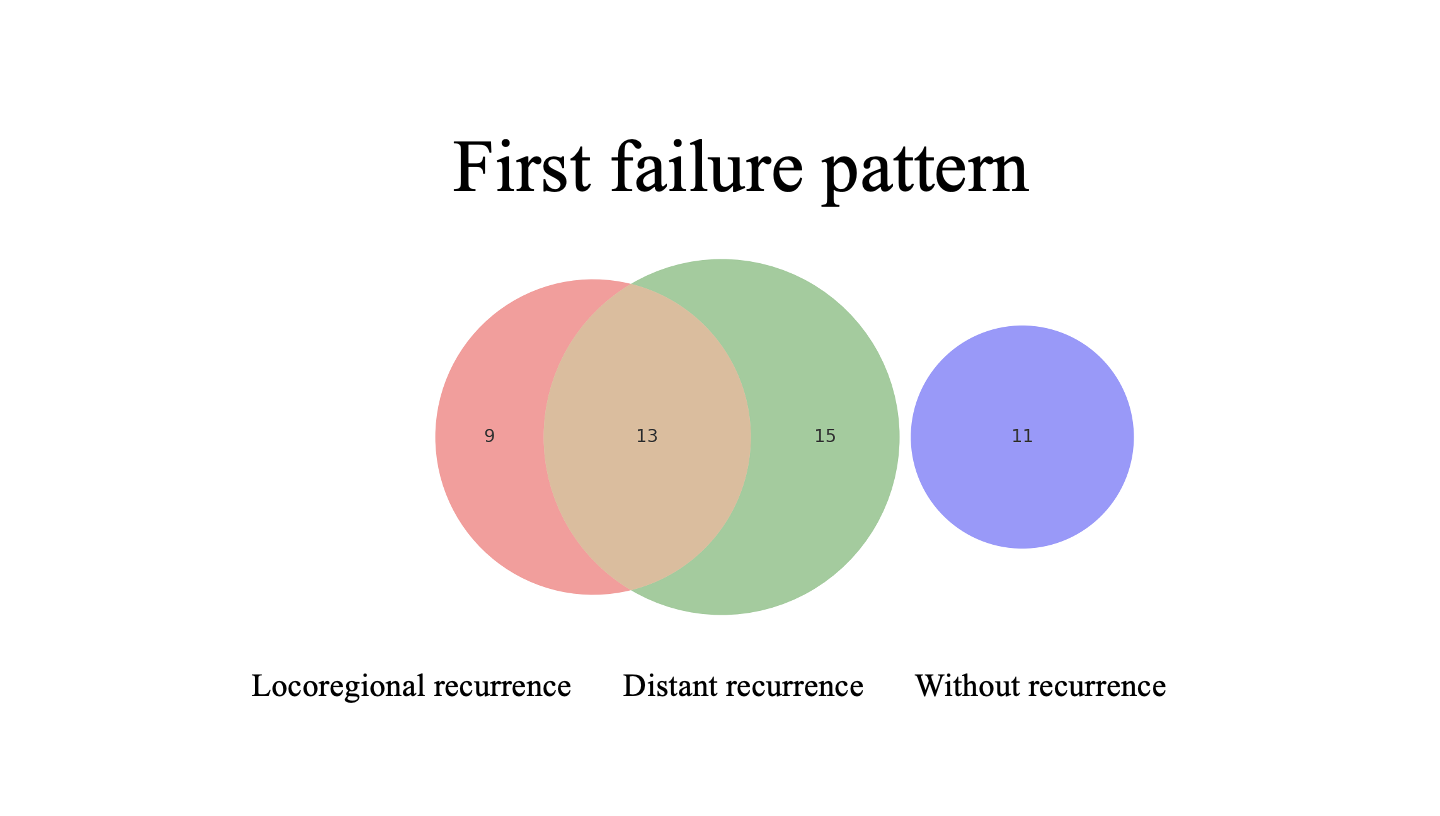

Supplement: Supplementary file 1 [file DataSheet1.docx]
