# Supplementary material for: Impact of smoking on immune feature and prognosis in unresectable stage III anaplastic lymphoma kinase positive non-small-cell lung cancer
Source: Front Oncol. 2026 Jan 23;15:1594479. doi: 10.3389/fonc.2025.1594479 (PMC12875992; doi:10.3389/fonc.2025.1594479)
Supplement: Supplementary file 2 [file DataSheet2.docx]

Supplementary Table S1. Baseline characteristics

|  | **Chemoradiation therapy** | **Systemic therapy** | **p.overall** |  |
| --- | --- | --- | --- | --- |
|  | ***N=16*** | ***N=32*** |  |  |
| Age (range) | 53.2 (33-75) | 56.2 (39-80) | 0.408 |  |
| Sex: |  |  | 0.759 |  |
| Woman | 9 (56.2%) | 15 (46.9%) |  |  |
| Man | 7 (43.8%) | 17 (53.1%) |  |  |
| ECOG PS: |  |  | 0.469 |  |
| 0 | 5 (31.2%) | 15 (46.9%) |  |  |
| 1 | 11 (68.8%) | 17 (53.1%) |  |  |
| Smoking: |  |  | 0.473 |  |
| No | 7 (43.8%) | 19 (59.4%) |  |  |
| Yes | 9 (56.2%) | 13 (40.6%) |  |  |
| Stage: |  |  | 0.195 |  |
| IIIA | 5 (31.2%) | 7 (21.9%) |  |  |
| IIIB | 11 (68.8%) | 19 (59.4%) |  |  |
| IIIC | 0 (0.00%) | 6 (18.8%) |  |  |
| Histological type: |  |  | 1.000 |  |
| Adenocarcinoma | 15 (93.8%) | 30 (93.8%) |  |  |
| Non-adenocarcinoma | 1 (6.25%) | 2 (6.25%) |  |  |
| EML4-ALK variants.: |  |  | 0.340 |  |
| V3 | 4 (25.0%) | 10 (31.2%) |  |  |
| Others | | 7 (43.8%) | 7 (21.9%) |  |
| Unknown | | 5 (31.2%) | 15 (46.9%) |  |

Abbreviation: ECOG PS, Eastern Cooperative Oncology Group performance status; *ALK*, anaplastic lymphoma kinase; TKIs, tyrosine kinase inhibitors.

Supplementary Table S2. Pattern of recurrence

| **Pattern of recurrence** | **Chemoradiation therapy**  **(*N=16*)** | **Systemic therapy**  **(*N=32*)** | ***p*** |
| --- | --- | --- | --- |
| Recurrence: |  |  | 0.075 |
| Without recurrence | 6 (37.6%) | 6 (18.8%) |  |
| Locoregional recurrence | 1 (6.25%) | 8 (25.0%) |  |
| Distant recurrence | 7 (43.8%) | 7 (21.9%) |  |
| Locoregional and distant recurrence | 2 (%) | 11 (34.4%) |  |
| Sites of first metastasis* |  |  | 0.849 |
| Brain | 5 (35.7%) | 9 (28.1%) |  |
| Liver | 3 (21.4%) | 3 (9.4%) |  |
| Bone | 1 (7.1%) | 3 (9.4%) |  |
| Others | 2 (14.3%) | 2(14.3%) |  |

* Some patients relapsed with multiple sites initially.

| **Pattern of recurrence** | **Chemoradiation therapy**  **(*N=16*)** | **Systemic therapy**  **(*N=32*)** | ***p*** |
| --- | --- | --- | --- |
| Locoregional recurrence: |  |  | 0.018 |
| Yes | 3 (18.8%) | 19 (59.4%) |  |
| No | 13 (81.2%) | 13 (40.6%) |  |
| Distant recurrence |  |  | 1.000 |
| Yes | 9 (56.2%) | 18 (56.2%) |  |
| No | 7 (43.8%) | 14 (43.8%) |  |

| **Pattern of recurrence** | **Never-smoker**  **(*N=26*)** | **Smoker**  **(*N=22*)** | ***p*** |
| --- | --- | --- | --- |
| Locoregional recurrence: |  |  | 0.735 |
| Yes | 13 (50%) | 9 (40.9%) |  |
| No | 13 (50%) | 13 (59.1%) |  |
| Distant recurrence |  |  | 0.511 |
| Yes | 13 (50%) | 14 (63.6%) |  |
| No | 13 (50%) | 8 (36.4%) |  |
